# Supplementary material for: A pistil-expressed pectin methylesterase confers cross-incompatibility between strains of Zea mays
Source: Nat Commun. 2019 May 24;10:2304. doi: 10.1038/s41467-019-10259-0 (PMC6534598; doi:10.1038/s41467-019-10259-0)
Supplement: Supplementary file 1 — Supplementary Information [file 41467_2019_10259_MOESM1_ESM.pdf]

A Pistil-Expressed Pectin Methylesterase Confers Cross-Incompatibility Between Strains of *Zea mays*

Lu, et al.

Supplementary Information

GCCAGCGAAGACGCGCACAAAGGTAAGTGTGTAAGGTTAGGAGCCAGTGTACCATCACATCAGA
TCAGATCGATCGCAGCTTATAGGGGATGGTAGGCGGCGTGAGGAGGTGCGGCCTGGGCCTGGCC
ATGGCGGTGGCCCTGCTGCTCGCTGCGCTGGTTGTCGTTGCAAGCGGCGGCGCGGAGACGAGGC
AGAAGCTGCCTGCTGGCAGTGGTAACGACGACGACCACGCAGCCGTTCTCAGCCGCCTGTCCAA
CGTCATTGATCCGCCGGGGAGCTGGCCTCCGCGTGCGGATGCTGTCGTGGCGAAGCGGTGCCGC
GGCGTCGCTGCTCCGCCGCCTTGCTACACCAGCATCCAGGCAGCCGTGGATCACGCACCAGCAC
CACAAGAAGCCGAGGAGGTGGAGGACAAGTACGTCGTGCATGTGCTCGCTGGCGTCTACGACGA
GACCGTCAACATAACAAGAAGGAACGTGATGCTGATCGGCGATGGGGTCGGTGCCACCGTCATC
ACGGGGAACAAGAGTAATGCAACAGGCGTCCACATGGACATGACGGCGACAGTGAgtgagtaac
tgtgccgatcgatatttcactgcagttgagctgtcatgcatgcatactgtaaatcagaacagaa
ctattaatttgctgctgctgctgcagATGCCTTGGGTCACGGCTTCATAGCACAGAACCTGACA
ATCAGAAACACGGCAGGGCCGGAAGGCAGGCAGGCCGTGGCGCTAAGGTCAAATTCGAACAAGT
CGGTCGTCTACTGGTGCAGCATTGAAGGTCATGAGGACACCTTGTACGTGGAGAACGGGATCCA
GTTCTACCTGCAGACCTCGATCTGGGGCACCGTGGACTTTGTGTTTGGCAATGCCCAGGCCATG
TTCCAGAGCTGCGCGCTGCTGGTGCGCCGCCCACCGAAAGGCAAGCACAATGTGCTGACGGCCC
AGGGCTGCAACAACGCAAGCCGCGAGTCCGGCTTCTCGTTCCACATGTGCACCGTGGAAGCCGC
GCCGGGCGTGGACCTCGACGGCGTGGAGACCTACCTCGGCCGCCCCTACAGGAACTTCTCCCAC
GTCGCCTTCATCAAGTCGTATCTCAGTCGCGTGGTCAGCCCCAACGGCTGGGTCGCGTGGAACA
AGAACAAGGTCGTCGAGGATACCACCCGGACCATCTTATACCTGGAATACGGCAACGACGGCGC
CGGTGCCGACACAGCCGGCCGCGTCAAGTGGCCGGGCTTCCGCGTCCTCAACACCGACGACGAG
GCGATCGCGTACACGGCGGACACGTTCATCAACGCGAGCAAGTGGGTCCCTGAGCCTATCCAGT
ACGTCCACACCCTCGGCACGGCGCCGCCGCCGCGCGCCTGACGATGTTGTACTACGCAGGCT

Supplementary Figure 1. Sequence of *Tcb1-f/PME38*. Exon sequence is shown in uppercase, and intron sequence is shown in lowercase. The coding sequence is underlined.

a

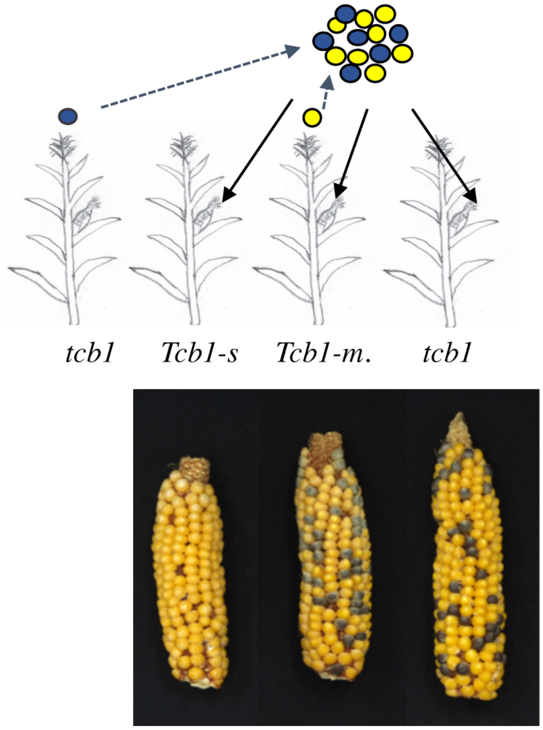

b

Supplementary Figure 2. Mix pollination testing the spontaneous *Tcb1-m* plant. a. scheme of the experiment: Details in "Method" section. b. ears from the three pollen receivers for *Tcb1-m* plant test. Pollen from the *Tcb1-m* plant successfully fertilized the *Tcb1-s* ear and produced yellow kernels (left ear in b), while the ears from *Tcb1-m* plant indeed had lost the barrier to block maize pollen as shown by the purple kernels produced by *tcb1* pollen on the ears (middle ear in b).

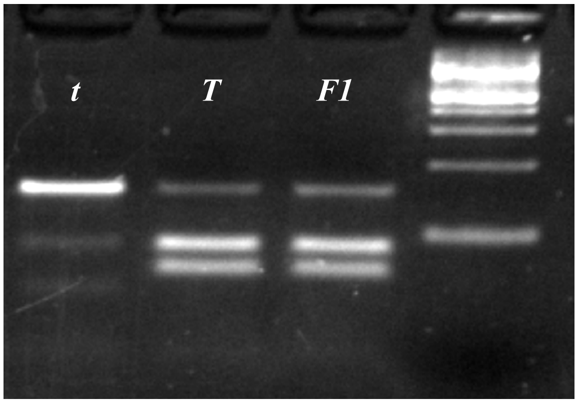

Supplementary Figure 3. A dCAPS marker to test presence/absence of *Tcb1-f/PME38* in recombinants from the mapping population. The Marker was designed in the way that only the PCR amplicon from the *Tcb1* genomic DNA (*T*), but not the unspecific PCR product amplified from the maize gnomic DNA (*t*) would be cut by the enzyme Hae III. after PCR and enzyme digestion.

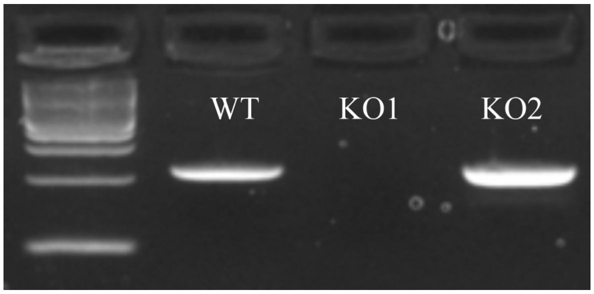

Supplementary Figure 4. PCR detection of *Tcb1-f/PME38* in the two *tcb1-f(KO)* mutants. PCR primers spanning the possible mutation site in the *tcb1-f(KO1)* was designed and tested on the *Tcb1-s* (WT), *tcb1-f(KO1)* and *tcb1-f(KO2)*. Using *tcb1-f(KO1)* genomic DNA as template failed to produce amplicon, while *Tcb1-f/PME38* can be detected in the *tcb1-f(KO2)* mutant.

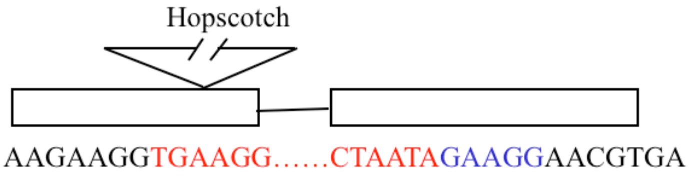

Supplementary Figure 5. Identification of the mutation in the *tcb1-f(KO1)* mutant. Whole genome resequencing showed a Hopscotch retrotransposon insertion in the first exon. PCR primers based on the gene and the retrotransposon were used to confirm the insertion and the border sequences. Black bases, *Tcb1-f/PME38* gene sequence; Red bases, retrotransposon sequence; Blue bases, bases duplicated from the left border leading the retrotransposon sequence.

```
Col104-4a parviglumis   ACAGTGAGTGAGTAACTCTGCCGATCGATATTTCACTGCAGTTGAG…
Col109-4a mexicana      ACAGTGAGTGAGTAACTCTGCCGATCGATATTTCACTGCAGTTGAG…
Col48703  mexicana      ACAGTGAGTGAGTAACTGTGCCGATCGATATTTCACTGCAGTTGAG…
Col207-5d mexicana      ACAGTGAGTGAGTAACTGTGCCGATCGATATTTCACTGCAGTTGAG…
Maiz Dulce              ACAGTGAGTGAGTAACTGTGCCGATCGATATTTCACTGCAGTTGAG…
                        ***************** ****************************
```

Supplementary Figure 6. Partial DNA alignment of the *Tcb1-f/PME38* gene intron between the different *Tcb1-s* lines. Collections 109-4a, 48703 and 207-5d, ssp. *mexicana* teosintes; Collection104-4a, ssp. *parviglumis* teosinte; DGF1222, a line derived from a traditional Maiz Dulce sweetcorn variety from Mexico (*13*).

```
Tcb1-f     MVGGVRRCGLGLAMAVALLLAALVVVASGGAETRQKLPAGSGNDDDHAAVLSRLSNVIDP
ZmPME3     MVGGVRRCGLGLAMAVALLLAALVVVASGGAEMRQKLPAGSGNDDDHAAVLSRLSNVIDP
           ******************************** ***************************

Tcb1-f     PGSWPPRADAVVAKRCRGVAAPPPCYTSIQAAVDHAPAPQEAEEVEDKYVVHVLAGVYDE
ZmPME3     PGSWPPRADAVVAKRCGGVAAPPPCYTSIQAALKAASAPQEAEEVEDKYVVHVLAGVYDE
           **************** ***************:. * ***********************

Tcb1-f     TVNITRRNVMLIGDGVGATVITGNKSNATGVHMDMTATVNALGHGFIAQNLTIRNTAGPE
ZmPME3     TVNITRRNVMLIGDGVGATVITGNKSNATGVHMDMTATVNALGHGFIAQNLTIRNTAGPD
           ***********************************************************:

Tcb1-f     GRQAVALRSNSNKSVVYWCSIEGHEDTLYVENGIQFYLQTSIWGTVDFVFGNAQAMFQSC
ZmPME3     GRQAVALRSNSNKSVVYCCSIEGHEDTLYVENGIQFYLQTSIWGTVDFVFGNAQAMFQSC
           ***************** ******************************************

Tcb1-f     ALLVRRPPKGKHNVLTAQGCNNASRESGFSFHMCTVEAAPGVDLDGVETYLGRPYRNFSH
ZmPME3     ALLVRRPPKGKHNVLTAQGCNNASRESGFSFHMCTVEAAPGVDLDGVETYLGRPYRNFSH
           ************************************************************

Tcb1-f     VAFIKSYLSRVVSPNGWVAWNKNKVVEDTTRTILYLEYGNDGAGADTAGRVKWPGFRVLN
ZmPME3     VAFIKSYLSRVVSPNGWVAWNKNKVVDDTTRTILYLEYGNDGAGADTAGRVKWPGFRVLN
           **************************:*********************************

Tcb1-f     TDDEAIAYTADTFINASKWVPEPIQYVHTLGTAPPPRA
ZmPME3     TDDEAIAYTADTFINASKWVPEPIQYVHTLGTAPPPRA
           **************************************
```

Supplementary Figure 7. Alignment of *Tcb1-f/PME38* and ZmPME3 proteins with Clustal Omega.

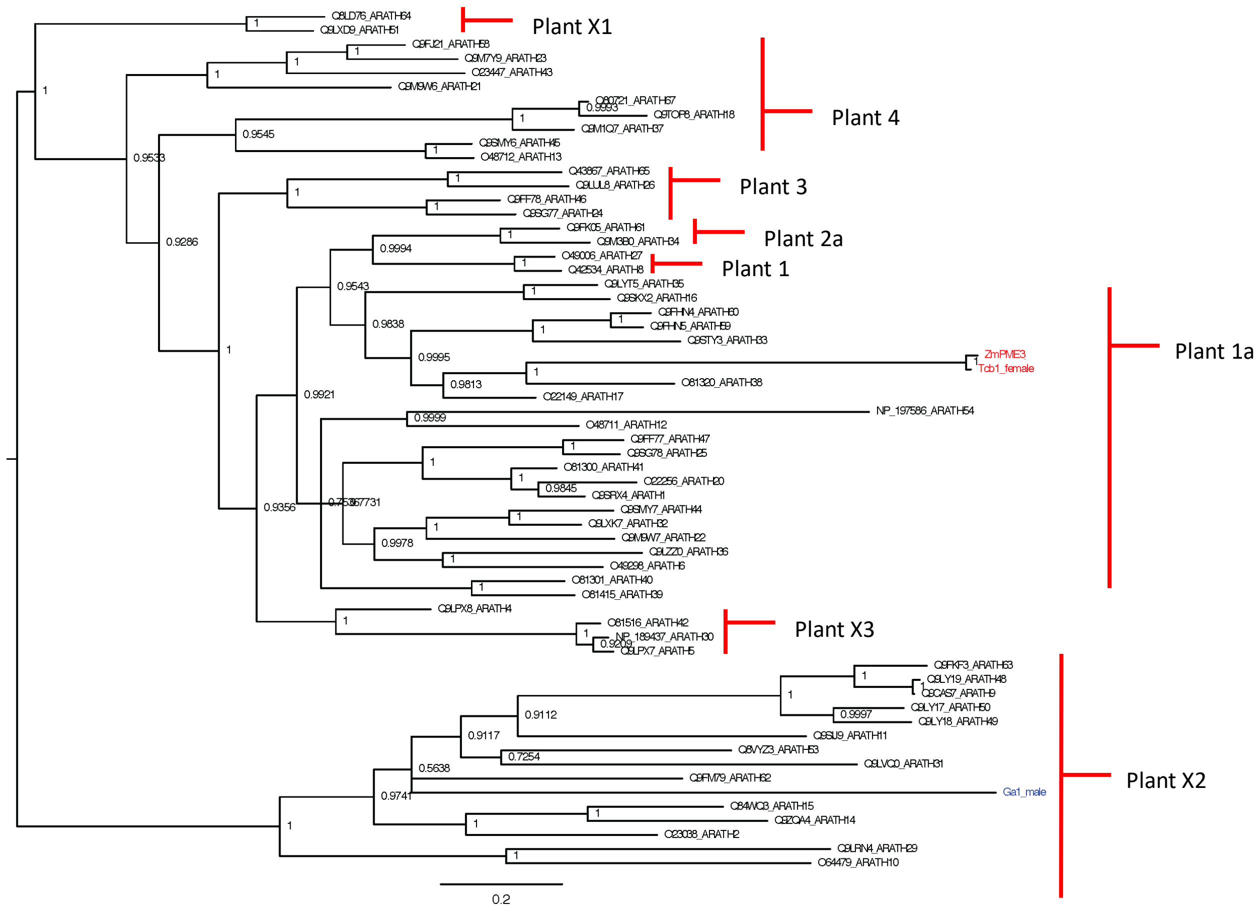

Supplementary Figure 8. Phylogenic tree of mature PME enzymes (predicted pre and pro domains removed) of Arabidopsis PME proteins and predicted PME proteins encoded by cross-incompatibility loci of *Zea mays*.

Supplementary Table 1. Haplotypes of the *tcb1* locus

Haplotype	Gene Content	Female Barrier	Pollen Function	Principal source
Tcb1-s	*Tcb1-f(PME38) Tcb1-m*	+	+	Teosinte
Tcb1-f	*Tcb1-f(PME38)*	+	-	Recombination
Tcb1-m	*Tcb1-m*	-	+	Teosinte
tcb1	-	-	-	Maize

Supplementary Table 2. PCR primers for mapping *Tcb1-s* relative to the maize B73 reference genome

Marker	Primer sequences	Marker types	Note
053600	FP: AGCAGGTGCCGCCCG T<br>RP: AAGCGAGGGTTTGCTCGAT	Indel	*Tcb1-s* left border
444073 up8	FP: GAGCAGGGTCAGCTGGAAGGAAC<br>RP: GGCATCTCTTGCTTGGCGGGC	dCAPS	*Tcb1-f* left border
Gene 0.1 up	FP: GATAAGTTTGCAAGTGGCCCATCA<br>RP: AGAGATGTTCTGCAATGGGCCTA	Indel	*Tcb1-m* right border
106164	FP: GGCTGAAGCAGCAGAGCCATCCTAA<br>RP: CTGTGTGTGGGATCGGATCCCTA	Indel	*Tcb1-s* right border

Supplementary Table 3. PCR primers for qRT-PCR	
Gene	Primer sequences
Tcb1-f/PME38	Forward: GCCGCCCCTACAGGAACTTC
	Reverse: GGTGGTATCCTCGACGACCTTG
Tubulin	Forward: ACGAGTTCCAGACCAACCTG
	Reverse: AGAGAGCTGCTCGTGGTAGG
